# High aerodynamic lift from the tail reduces drag in gliding raptors

**DOI:** 10.1242/jeb.214809

**Published:** 2020-02-10

**Authors:** James R. Usherwood, Jorn A. Cheney, Jialei Song, Shane P. Windsor, Jonathan P. J. Stevenson, Uwe Dierksheide, Alex Nila, Richard J. Bomphrey

**Affiliations:** 1 Structure and Motion Laboratory, The Royal Veterinary College, North Mymms, Hatfield, Herts AL9 7TA, UK; 2 School of Mechanical Engineering, Dongguan University of Technology, Dongguan, Guangdong, China; 3 Department of Aerospace Engineering, University of Bristol, Queens Building, University Walk, Bristol BS8 1TR, UK; 4 LaVision GmbH, Anna-Vandenhoeck-Ring 19, 37081 Göttingen, Germany; 5 LaVision UK Ltd, 2 Minton Place, Victoria Road, Bicester, Oxon OX26 6QB, UK

**Keywords:** Bird, Reynolds number, Stability, Particle tracking velocimetry, Flight

## Abstract

Many functions have been postulated for the aerodynamic role of the avian tail during steady-state flight. By analogy with conventional aircraft, the tail might provide passive pitch stability if it produced very low or negative lift. Alternatively, aeronautical principles might suggest strategies that allow the tail to reduce inviscid, induced drag: if the wings and tail act in different horizontal planes, they might benefit from biplane-like aerodynamics; if they act in the same plane, lift from the tail might compensate for lift lost over the fuselage (body), reducing induced drag with a more even downwash profile. However, textbook aeronautical principles should be applied with caution because birds have highly capable sensing and active control, presumably reducing the demand for passive aerodynamic stability, and, because of their small size and low flight speeds, operate at Reynolds numbers two orders of magnitude below those of light aircraft. Here, by tracking up to 20,000, 0.3 mm neutrally buoyant soap bubbles behind a gliding barn owl, tawny owl and goshawk, we found that downwash velocity due to the body/tail consistently exceeds that due to the wings. The downwash measured behind the centreline is quantitatively consistent with an alternative hypothesis: that of constant lift production per planform area, a requirement for minimizing viscous, profile drag. Gliding raptors use lift distributions that compromise both inviscid induced drag minimization and static pitch stability, instead adopting a strategy that reduces the viscous drag, which is of proportionately greater importance to lower Reynolds number fliers.

## Abstract (precis)

**Summary:** Aerodynamic lift from gliding hawk and owl tails, revealed by tracking helium bubbles, is inconsistent with passive stability or minimizing induced drag, but indicates a role in reducing viscous drag.

## INTRODUCTION

Bird tails clearly perform many roles, both in terms of display and as aerodynamically active surfaces. The potential aerodynamic roles performed by bird tails can be divided into manoeuvrability, stability, lift production and drag reduction through a variety of mechanisms (Thomas, 1996; Maybury and Rayner, 2001; Huyssen et al., 2012). These functions often have opposing demands: it is difficult to enhance both manoeuvrability and static stability; lift production often comes at the cost of increased drag.

Conventional aircraft tails act as rudders, elevators and stabilizers, providing moments about the centre of mass to initiate and maintain turns, and restoring moments that correct perturbations from trimmed, level flight. Bird tails have a quite different form, lacking the vertical fin of typical aircraft. Further, tails are not a requirement for competent, manoeuvrable flight for flapping animals: birds without tails are still able to achieve some – albeit ungainly – level of control, and many bats are functionally tailless. Flying animals differ markedly from traditional fixed wing aircraft in a number of ways: they flap, they have rapid sensing and complex control capability, and they are, at least in some gliding cases, aerodynamically unstable (Durston et al., 2019; Durston, 2019). They are also smaller and slower, so potentially operate under quite different aerodynamic regimes. How, then, should the aerodynamic role of the bird tail be understood?

**Fig. 1.**
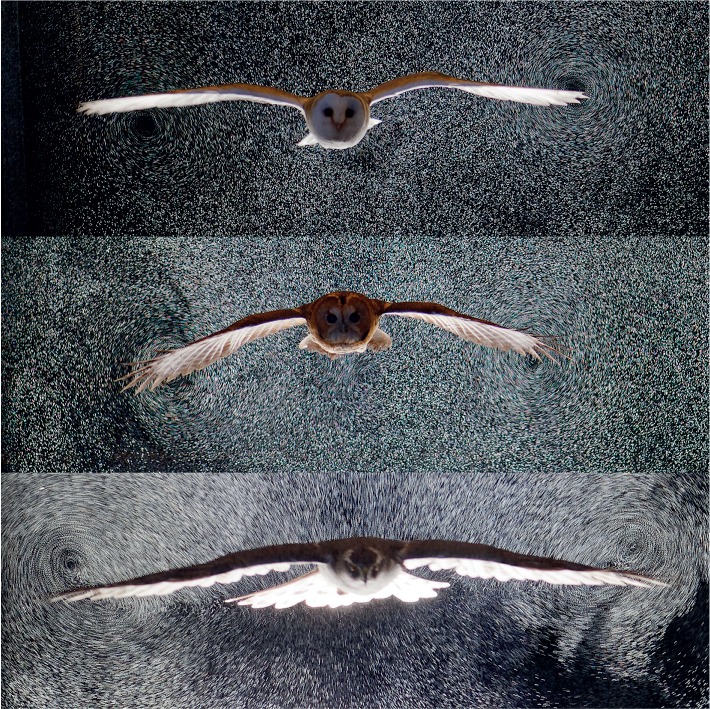
**Air motions caused by gliding raptors visualized with bubbles.** Photographs of a gliding barn owl (top), tawny owl (middle) and goshawk (bottom) as, or narrowly after, they passed through a 0.1 m light sheet seeded with neutrally buoyant 0.3 mm soap bubbles. See also Movie 1.

In order to explore the aerodynamics of gliding in a range of raptors, we measured the flow field through particle tracking of neutrally buoyant 0.3 mm helium bubbles (Fig. 1; Movie 1). Application of automated Lagrangian particle tracking velocimetry (see Movie 2) to the study of bird flight is novel, though seeding the air with helium bubbles builds upon the early studies of animal flight (Spedding et al., 1984; Spedding, 1987); and wakes have been measured using smoke and particle image velocimetry for a range of considerably smaller flapping (Spedding et al., 2003; Warrick et al., 2005; Van Griethuijsen et al., 2006; Tobalske et al., 2009; Altshuler et al., 2009; Johansson et al., 2018) and gliding (Henningsson and Hedenström, 2011; Henningsson et al., 2014; KleinHeerenbrink et al., 2016) birds.

**Fig. 2.**
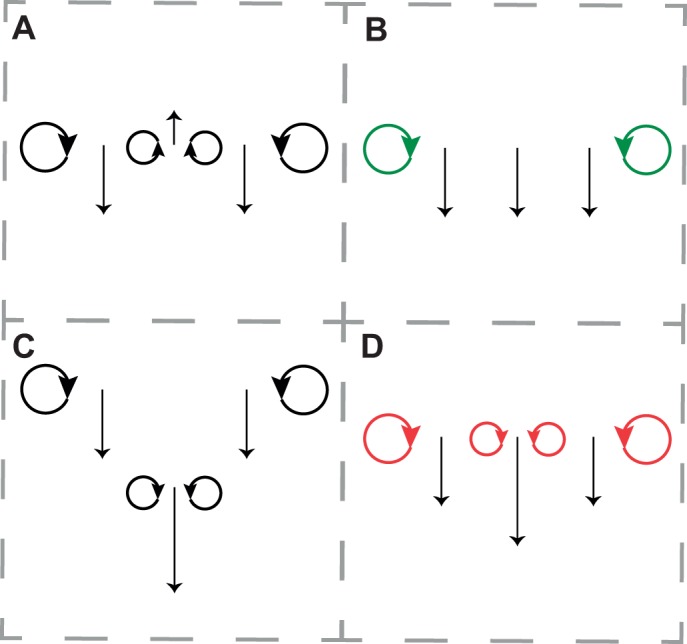
***Post hoc* hypotheses for competing models of tail function in steady gliding.** Negative lift from the tail (A) might improve pitch stability; induced drag might be low (B) if the tail counteracted loss of lift over the body; or induced drag might be reduced through biplane aerodynamics (C). A step increase in lift over the body/tail section would be evident from trailing vortices following behind the tail of the same sense as those following the wingtips on the same side (D), associated with an increase in downwash velocity, and would be inconsistent with simple pitch stability or minimization of induced drag.

Following initial inspection of the bubble motions, interpretations for various wake structures were developed. These can be presented here as hypotheses, though their *post hoc* nature should be acknowledged. The rotational sense and initial relative position of trailing vortices behind wing tips and body/tail section distinguish certain potential tail actions (Fig. 2). Many traditional aircraft make use of negative lift from the tail, resulting in ‘longitudinal dihedral’ to improve stability in pitch; this would result in upwash from the tail, and trailing vortices following the wing/body of opposite sense to those following the wing tips on the same side (Fig. 2A). A tail/body section that does not disrupt the downwash would result in the absence of trailing vortices behind the tail (Fig. 2B). Drag reduction through biplane aerodynamics (Thomas, 1996) would require wing tip and body/tail trailing vortices of the same sense each side, but with vertical offset (Fig. 2C). Vortices with the same sense each side but without the offset (Fig. 2D) indicate an increased lift across the body/tail section, detrimental to induced drag minimization but potentially consistent with drag reduction at low Reynolds numbers.

## MATERIALS AND METHODS

### Birds

Three captive and mature raptors were used in this study: a female barn owl [*Tyto alba* (Scopoli 1769)], a male tawny owl (*Strix aluco* Linnaeus 1758) and a female northern goshawk [*Accipiter gentilis* (Linnaeus 1758)]. All individuals were trained to fly between handlers on command and were experienced at operating in brightly illuminated and unusual environments, such as film sets. Work was approved by the Ethics and Welfare Committee of the Royal Veterinary College (URN 2018 1836-3).

### Experimental setup

Experiments were conducted within a purpose-built indoor flight corridor at the Royal Veterinary College (Hatfield, UK). The corridor was constructed to (1) prevent ambient air flow from introducing noise to the measured flow fields; (2) prevent dispersal of the helium-filled soap bubble tracer particles; and (3) create a dark background for maximizing image signal (bubbles) to noise (background). The corridor was roofed and black on all inner surfaces. It was contained within a larger room, with the end of the flight path open to the room, allowing ambient light to illuminate the receiving handler. The measurement volume was not illuminated until after the birds entered it; otherwise, birds reacted to the illuminated volume of bubbles as if it were a wall. The corridor was approximately 1.8 m wide×1.8 m tall×14 m long. Results from three trials each for the three birds are reported here.

For each trial, bubbles were injected into the volume and allowed to quiesce prior to the flight. Bubbles were generated with 40 nozzles, and a fluid supply unit (LaVision GmbH) regulated soap, helium and air content to maintain neutral buoyancy. Bubbles were approximately 300 µm in diameter and, because of their large size and light scattering properties, were approximately 10,000 times brighter than standard-use aerosol particles for particle image velocimetry (Caridi, 2018), allowing LED lights to provide sufficient illumination, rather than high-power laser light sources that could potentially be damaging to birds' vision.

During each recorded flight, the bird flapped along the corridor, gaining speed before entering a smooth, steady glide just before the measurement volume. Initiation of LED illumination of the measurement volume was controlled using a hand trigger.

### Imaging

The measurement volume was constrained to the region illuminated by the LEDs. Four high-power LED units (LED-Flashlight 300, LaVision GmbH) illuminated the bubbles. Each LED unit consisted of an array of 72 CoB LEDs arranged over an active area of 300×100 mm^2^, with each CoB LED subunit focused with a lens to a divergence of 10 deg. Four units were placed side by side pointing upward, and a concave mirror on the corridor roof reflected light back down. Because of divergence and reflection, the four LED light units covered an effective measurement region slightly greater than 1.2 m×0.1 m. LEDs strobed in synchrony with the video frame capture and with the same 10:1 duty cycle, thereby maximizing useful illumination while minimizing electrical power demand and the brightness perceived by the birds.

The illuminated volume was captured using four high-speed cameras recording at 700 Hz (VEO 640L, Phantom Inc.; and Fastcam SA3, Photron Inc.). Cameras were positioned principally along the flight path, facing the bird as it entered the illuminated volume. Cameras and LED lights were synchronized and controlled with a timing unit (PTU X, LaVision GmbH). Further cameras (Nikon D3, Nikon Corporation; Red Epic Dragon, Red.com, LLC, at 120 Hz,) situated behind the receiving handler provided context images (Fig. 1) and video (Movie 1) for a subset of trials.

### Camera calibration

A two-stage iterative camera calibration process was used, followed by the unusual step of estimating projected bubble shape as a function of position. We first calibrated using a standard target (a dot grid), then improved the calibration by minimizing reprojection error of images of bubbles at moderate seeding density. Because imaging is diffraction limited, bubbles project onto the sensor as diffraction-induced airy disc patterns, with disc shape a function of position due to optical aberrations. Using the same bubble images at moderate seeding density, an optical transfer function was estimated for the modified airy disc shape as a function of position, which improved our capacity to resolve bubble location, and better accounted for overlapping bubbles.

### Particle tracking: ‘shake-the-box’

The ‘shake-the-box’ (STB) algorithm (Schanz et al., 2013, 2016) is a 4D particle tracking algorithm that identifies particle positions in 3D space by triangulation and follows individual particles over time. The output from STB consists of individual particle tracks, from which velocities and accelerations are derived. This contrasts to the output from Tomo-PIV, which is a regular grid of velocity vectors. After a bubble is located in space, its projection onto the image is subtracted to yield residual images showing only the remaining particles yet to be located. The STB algorithm makes use of the particle track information from previous time steps to predict the new particle position in subsequent time steps. This predicted 3D position is prioritized in the search for matching 2D particle images on the camera frames. Finally, this particle position is subsequently ‘shaken’ to maximize the match with the camera images.

### Image processing

Prior to volume self-calibration, and in addition to all dataset processing, image sets were pre-processed to optimize image quality. A combination of time-based and spatial filters was used to remove image artefacts such as background noise and image noise. The time-based filter removes stationary artefacts by means of subtracting the minimum recorded value at each pixel from a set of images for a camera. Spatial image filters reduce image noise and normalize image intensity. Image noise was reduced using a sliding window to subtract minimum intensity contained in a 7×7 pixel window, larger than twice the particle image diameter (which here was on average 3–4 pixels). Particle intensity, which varies as a result of scattering angle, was standardized across the image by normalizing the values using a local average based on a 300×300 sliding pixel window.

### Vortex structure identification using the *Q*-criterion

**Fig. 3.**
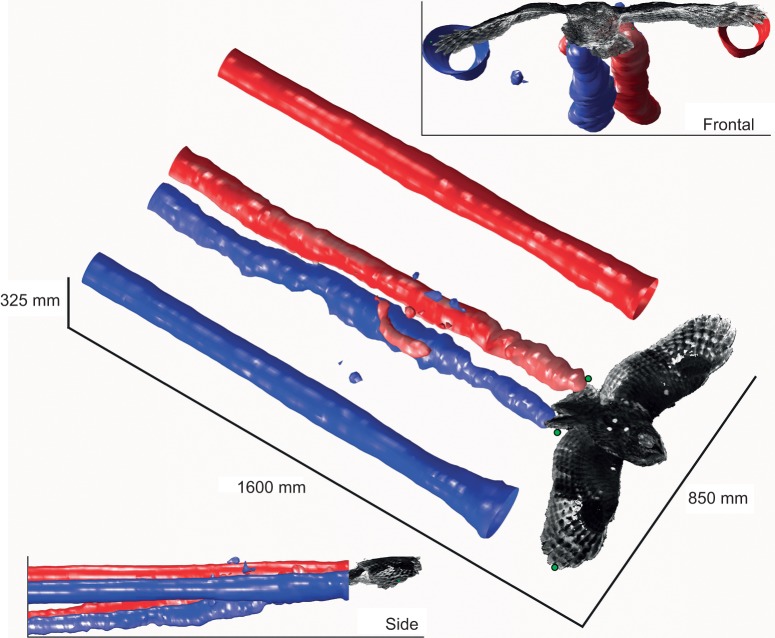
**An example reconstruction** **of vortex structures behind a gliding tawny owl.** Isosurfaces of the wake displayed using the *Q*-criterion highlight two discrete pairs of trailing vortices: an outer pair behind the wing tips and a narrower pair trailing the body/tail section (blue – clockwise facing the bird; red – anticlockwise). A representative owl surface geometry is shown as a 3D point cloud derived from video stereogrammetry of previous glides, planform matched and orientated with four landmarks (green dots) at the wing and tail-tips measured in the particle tracking velocimetry (PTV) trials. Reconstructions for all trials are shown in Fig. 4.

**Fig. 4.**
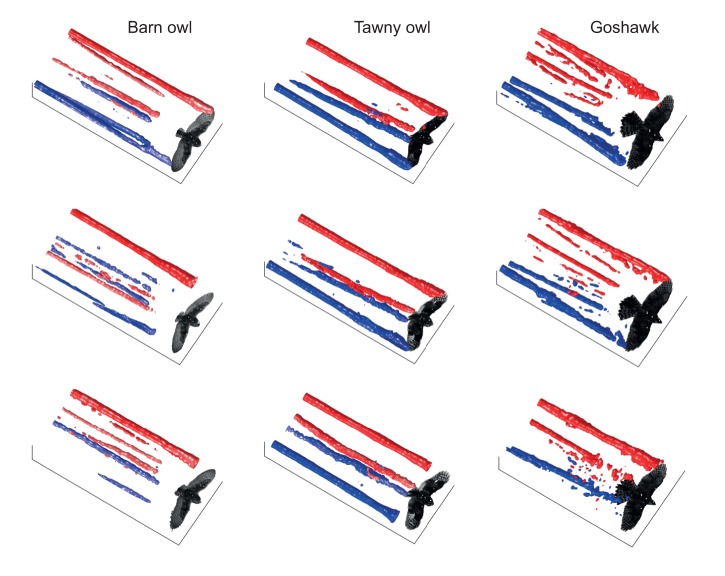
**Vortex structures behind a gliding barn owl, tawny owl and goshawk measured with PTV of neutrally buoyant bubbles.** Two discrete pairs of trailing vortices were consistently observed: an outer pair behind the wing tips and a narrower pair trailing the body/tail section. Colours indicate vortex sense: blue – clockwise facing the bird; red – anticlockwise. Air between a pair of red/blue vortices is travelling downwards faster. The vortices behind the tail indicate a step increase in lift over the body/tail section.

The *Q*-criterion aims to capture the fluid ‘particles’ for which rotation predominates over shear strain, with the additional condition that pressure is lower than the ambient value (Jeong and Hussain, 1995). In our implementation, we considered the flow to be incompressible (Mach number ∼0.03), and solved the *Q*-value as:(1)
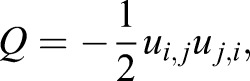
where *u_i_*_,*j*_ describes the partial derivative of the flow along axis *i*, taken in the *j* direction, and *i*, *j*=1,2,3 as in the Einstein summation. Critical *Q*-values were selected to highlight the dominant vortex structures (Figs 3 and 4).

### Downwash calculation

To compute downwash, particle velocities were placed into a uniform 3D grid using the Fine scale reconstruction (or VIC#) module in DaVis 10. Fine scale reconstruction is a PTV interpolation method similar to the ‘vortex in cell plus’ (VIC+) method which interpolates flow using the instantaneous spatial and temporal information from each bubble, linking the two with the Navier–Stokes equations (Schneiders and Scarano, 2016). The approach is grid based, and here we selected a 16×16×16 voxel window to form the grid. Window size was selected based on the observation that flow speed was maintained when compared with smaller windows, but with substantially less noise.

To estimate wake evolution, as in Figs 3 and 4, the middle, frontal plane for each time step in the flight direction was extracted and stacked. The time axis was converted to a spatial axis based on average forward flight speed, which was estimated from digitization of the birds passing through the volume.

### Bird planform

We could not comprehensively resolve bird planform from our camera views, but made use of relevant 3D reconstruction data collected from an earlier series of observations. To ensure appropriate planform selection, we digitized wing- and tail-tip position from images of the birds in the measurement volume, and selected planforms that best matched the spans and span ratio in this study. Planforms are from the same barn owl and goshawk individuals, but a different tawny owl. We then calculated planform from the boundary of the projected point clouds, from which chord profiles and derivative metrics were calculated (Table 1).

**Table 1.**
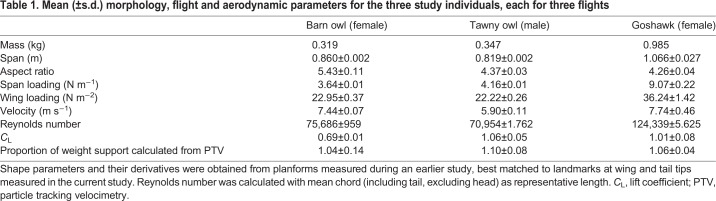
**Mean (±s.d.) morphology, flight and aerodynamic parameters for the three study individuals, each for three flights**

## RESULTS

Flights selected for analysis were steady, broadly level glides at relatively low speeds (Table 1). Motion of the seeding bubbles revealed trailing vortices in the wake of the wingtips, clearly visible in the photographs (Fig. 1) and movies (Movie 1). These vortices were tracked and quantified (Movie 2), and are displayed using isosurfaces of the wake *Q*-value (Figs 3 and 4). Trailing vortices behind the wing tips associated with downwash following the birds – and the momentum flux resulting in weight support – are not surprising, and entirely match expectations from aerodynamic theory and experience from aeronautics. What is more noteworthy is that discrete trailing vortices were also consistently observed in the wake behind the body and tail (Figs 1, 3 and 4).

## DISCUSSION

The trailing vortices following the tail, and the associated downwash near the bird centreline, demonstrate that the body/tail section produces greater aerodynamic lift per span than the wings. This positive lift is opposite to that required for tails producing stability through longitudinal dihedral: the tails of conventional, passively stable aircraft produce negative lift and accelerate air in the opposite direction – upwards – which would be associated with trailing vortices of the opposite sense.

If not used for passive pitch stability, it might be expected that the bird tails contribute to weight support during slow flight, and this is consistent with balancing of pitch moments in hawks (Tucker, 1992), visualization of gliding swift (Henningsson and Hedenström, 2011; Henningsson et al., 2014) and jackdaw (KleinHeerenbrink et al., 2016) wakes, and direct pressure measurements through pigeon tails (Usherwood et al., 2005). However, the observed trailing vortices behind the tail indicate that lift contribution of the central section is considerably in excess of simply filling in the lift distribution between the wings. The lift coefficients calculated for the tawny owl and goshawk were high for raptor wings (Withers, 1981; Van Oorschot et al., 2016), close to 1, so there is the possibility that tail lift is merely allowing slow gliding while preventing stall, analogous to the flaps deployed by landing aircraft (Pennycuick, 1975). However, the barn owl operated with a mean lift coefficient close to 0.7 – well below the maximum lift coefficient measured for isolated raptor wings (Withers, 1981; Van Oorschot et al., 2016) – yet also displayed the step increase in downwash behind the tail, meaning that a simple account based on stall avoidance is insufficient.

The apparently excessive aerodynamic lift produced by the body/tail is significant because it affects the drag experienced by the gliding bird. To understand its implications in terms of overall drag, we adapted classical approaches (Tucker, 1987; Spedding and McArthur, 2010) to model the drag *D* produced by wings of aspect ratio AR and area *S* through air of density ρ at flight speed *V* with wings at lift coefficient *C*_L_. In this presentation, total drag due to the wings can be separated into three components:(2)
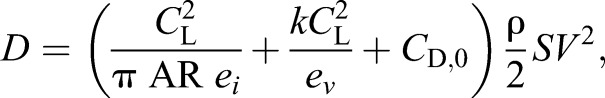
where *e*_i_ and *e*_v_ are inviscid and viscous efficiency factors, respectively. An *e* value of 1 is ideal, and the factors reducing efficiency from unity form the basis of the analysis developed here. The first term is the inviscid or induced drag coefficient – that associated with accelerating air downward in order to provide weight support. The second and third terms together combine to give the profile drag coefficient, with *C*_D,0_ the minimum drag coefficient (assumed here to occur close to zero lift). It is important to highlight that the second term increases with the square of lift coefficient, denoting the C-shape of a lift–drag polar for a generic pre-stall aerofoil (2D); the curvature of the polar relates to the constant *k* that expresses the quadratic rise of this drag term with lift (Spedding and McArthur, 2010), and tends to be more extreme at lower Reynolds numbers (Abbott and Doenhoff, 1949). This dependency on lift can present some confusion as it is sometimes convenient to combine it with the inviscid induced drag term (Houghton et al., 2016; Spedding and McArthur, 2010), which also varies with 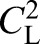. It is, however, a form of viscous drag and is therefore of proportionally greater magnitude at lower Reynolds numbers.

### Relating drag minimization predictions to downwash profiles

**Fig. 5.**
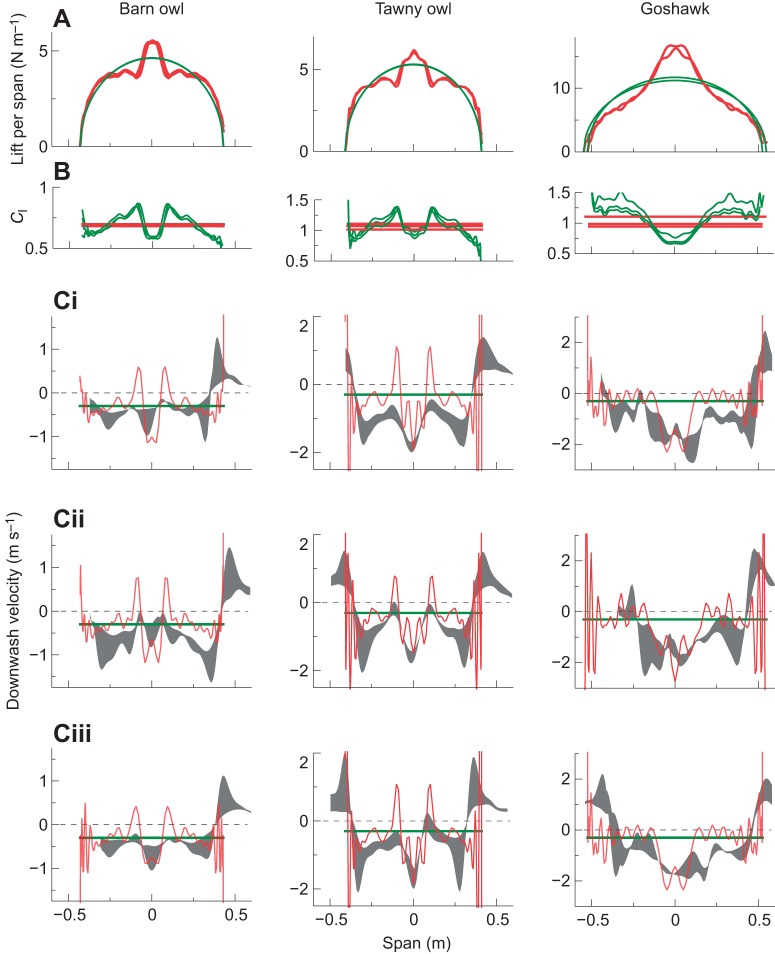
**Comparison of competing models of drag minimization.** Hypothetical spanwise lift profiles (A) and associated sectional lift coefficients (*C*_l_; B), and their modelled consequences in terms of downwash profiles (coloured lines) for three glides per species (Ci–iii). Green lines indicate the hypothetical inviscid or induced-drag minimizing case, with elliptical spanwise lift distribution, variable lift coefficient and constant downwash velocity across the span. Red lines indicate the theoretical viscous or profile-drag minimizing strategy, with lift distribution matching the chord profile of the wings/body planform resulting in a constant spanwise lift coefficient and – because the planform is not elliptical – varying downwash velocity. The deviation in planform from elliptical, largely due to the projecting central tail area, is evident from A, in which the loading profile is either elliptical or in direct proportion to chord (excluding the head). Grey shading indicates measurements spanning the maximum to minimum downwash velocities across horizontal transects of transverse planes after passage of the bird, located level with the wingtips, and 50 and 100 mm below the wingtips.

In order to compare the predictions from minimization of inviscid and viscous (or induced and profile) drag separately, the downwash profiles minimizing each were calculated and compared with measured profiles for gliding barn owl, tawny owl and goshawk (Fig. 5).

Inviscid or induced drag is classically minimized with an elliptical lift distribution across the span (Prandtl, 1921; Munk, 1923) (Fig. 5A, green lines), leading to a constant downwash velocity of sufficient magnitude to support body weight, but resulting in lift coefficients that vary across the planform (Fig. 5C, green lines). Viscous, profile drag, in contrast, is minimized (Fig. 5, red lines) if the lift coefficient is constant for every section, as, from Eqn 1:(3)
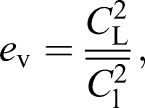
for wings of sectional lift coefficient *C*_l_ and near-constant aerofoil section shape*.* This requires that lift is evenly distributed across the planform area, and so spanwise lift profile matches the aerodynamic chord profile – in which case 
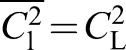
 and *e*_v_=1. Minimization of inviscid, induced drag and viscous, profile drag cannot both be met simultaneously without an elliptical planform.

Spanwise chord profiles matching the wing and tail spans of the measured glides were calculated from point clouds, excluding the head, from earlier glides using high-speed video photogrammetric methods, and were fitted with 50 Fourier terms to provide a close – though constrained to be symmetrical about the centre line – representation of the chord profile. This technique allows classical aerodynamic methods (Munk, 1923; Prandtl, 1921; Houghton et al., 2016, Phillips et al., 2019) to be applied to determine the associated downwash profiles given the assumption that profile drag is minimized if all sections operate at constant lift coefficient (and the lift coefficient is sufficient to support body weight).

### Derived downwash results and discussion

Downwash velocity fields for each trial were measured for a transverse plane closely after the passage of the tail trailing edge, but also dependent on good bubble seeding coverage. As these planes were not exactly at the ‘lifting line’ aerodynamic abstraction (a concept underlying the simplest 3D wing theory – Prandtl, 1921; see Abbott and Doenhoff, 1949), downward convection, though gradual (Figs 3 and 4), meant that no single horizontal transect across the plane provided an adequate measurement of downwash profile; instead, we show the range between maximum and minimum downwash values for transects at 0, 50 and 100 mm below the wingtips (Fig. 5).

**Fig. 6.**
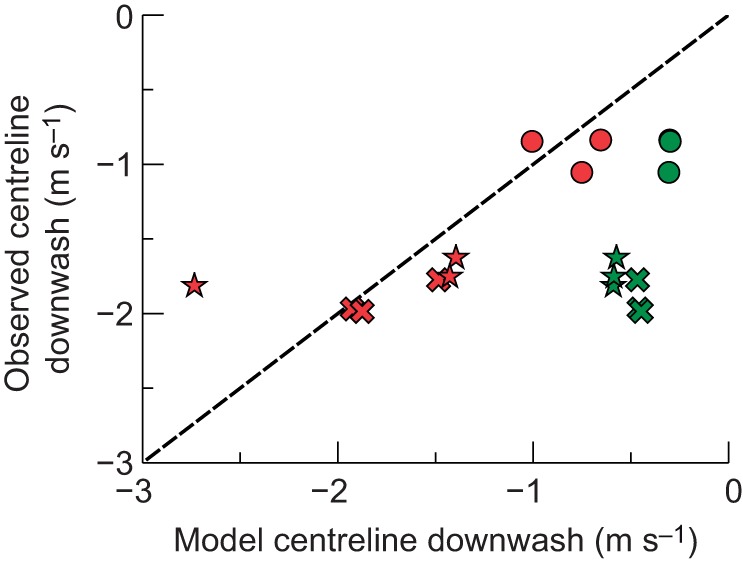
**Measured downwash quantitatively agrees with a significant role for viscous drag minimization and qualitatively refutes alternative hypotheses of tail function in gliding.** Measurements of downwash following the body/tail centreline section (A) (three species, three trials each) show close agreement (24% root mean square error, RMSE) with a profile drag minimizing (red) role for the tail; whereas, the induced drag minimizing (green) model consistently underpredicts downwash (247% RMSE). Treating each glide as an independent sample (while acknowledging the issues with this assumption), Mann–Whitney *U-*tests on the residuals indicate that the two models deviate from observation to different degrees (*P*<0.05): the induced drag minimizing model deviates significantly from observation (*P*<0.005) but the profile drag minimizing model does not (*P*=0.25). The profile drag minimizing, constant spanwise lift coefficient hypothesis with increased lift over and downwash behind the broader body/tail section is supported both qualitatively, with the presence of discrete tail tip vortices associated with positive lift (Figs 1, 3, 4; Movies 1, 2; contrast with Fig. 2), and quantitatively through downwash modelling. Circles: barn owl; crosses: tawny owl; stars: goshawk.

Downwash values at the centreline did not match the prediction of constant downwash from inviscid induced drag minimization. Instead, they provide a good quantitative match (Fig. 6) with predictions based on constant spanwise lift coefficient and minimized profile drag. The success of the second model, and contrast with aircraft-based postulations, may reflect both the relatively large contribution of viscous effects at the low Reynolds numbers (∼100,000) experienced by birds and a low cost to birds for their moderate deviation from perfect induced drag minimization. Indeed, using the constant-*C*_l_ theoretical downwash profiles, *e*_i_ is only reduced to 0.8–0.9.

We can therefore reject the action of the tail – at least under the conditions measured – as: (1) passive pitch stabilizer, which would require negative lift from the tail, upwash and associated trailing vortices of opposite sense from those we observed behind the body/tail (Fig. 2A); (2) downwash compensator, restoring lift lost over the body and minimizing inviscid induced drag (Huyssen et al., 2012), as this would result in constant downwash and only wingtip vortices being manifest in the wake (Fig. 2B); or (3) a functional biplane (Thomas, 1996) (Fig. 2C), because the wing and tail tips and their trailing vortices initially lie in the same horizontal plane. We found that the body/tail section contributes lift proportional to chord, thereby spreading the load across a greater surface and reducing the profile drag. We conclude, therefore, that the tail does not contribute to passive pitch stability with a longitudinal dihedral mechanism but, in addition to its role in moment generation when manoeuvring (e.g. Gillies et al., 2011), acts as an aerodynamic wing ‘flap’, expanding the aerodynamic planform area. However, whereas aircraft flaps are required for stall avoidance and increase drag, bird tails produce aerodynamic lift even when not near a stall limit, and act to reduce overall drag at low Reynolds numbers.

### Further caveats and comments

#### A note on passive longitudinal stability

We do not present here a full stability analysis for the birds of this study; this would require measurement or modelling of the inertial properties of each bird in gliding posture. See Durston (2019) for such an analysis of two raptors, which demonstrates a high degree of longitudinal instability. Positive lift from an aft aerofoil does not necessarily preclude the possibility that static longitudinal stability is obtained; indeed, this is a feature of certain aeroplane styles such as the ‘canard’ design, which has a smaller pair of wings ahead of the main, often delta, wing. However, the traditional aeroplane design appears to be a better initial analogue, with the larger lifting surface ahead of the smaller. In this case, a large upward lift from the tail is inconsistent with longitudinal static stability. The observed strong downwash and positive lift from the tail does therefore suggest that the tail is not contributing to static longitudinal stability, at least by the mechanism of longitudinal dihedral as exploited in traditional aeroplane designs.

#### A note on non-elliptical loading for induced drag minimization

While an elliptical loading distribution provides the theoretical minimum induced drag for a constrained wing span, other loading distributions are optimal given different constraints. Various structural, geometrical and weight considerations, along with passive yaw stability, may be important in aircraft design, leading to a range of non-elliptical loading distributions providing theoretical optima for minimizing induced drag (Prandtl, 1933; Phillips et al., 2019). The optimal loading distributions with such constraints tend to be more ‘bell shaped’, with a bias in loading towards central sections of the vehicle. However, the question of relevance in the current case is not ‘how can induced drag be minimized given certain constraints to do with stress, deflection or bending moment?’ but ‘how would induced drag be minimized given the wings available?’, i.e. given their maximum span. Induced drag is only reduced with bell-shaped loading distributions if the wing span is unconstrained. The spans of the birds in this study were certainly constrained, and so the theoretical minimum induced drag prediction remains that of elliptical spanwise loading and perfectly constant downwash velocity in the immediate wake. Despite this, the conceptual basis behind the advantages of bell-shaped loading distributions may have some relevance to the case of birds. High weight support by the central sections would indeed reduce the bending moment demanded at the wing roots – corresponding to torque around the shoulders – reducing at least some degree of muscle action and associated physiological costs. Bell-shaped loading distributions therefore have the potential to reduce the metabolic demands of gliding with a mechanism other than drag reduction. Consequently, while the viscous drag minimizing account proposed here provides a reasonable and quantitatively sufficient reasoning for the action of the tail during gliding, some alternative options cannot be rejected without further study.

## Supplementary Material

Supplementary information
