# Supplementary material for: High aerodynamic lift from the tail reduces drag in gliding raptors
Source: J Exp Biol. 2020 Feb 10;223(3):jeb214809. doi: 10.1242/jeb.214809 (PMC7033732; doi:10.1242/jeb.214809)
Supplement: Supplementary information [file jexbio-223-214809-s1.pdf]

SUPPLEMENTARY INFORMATION

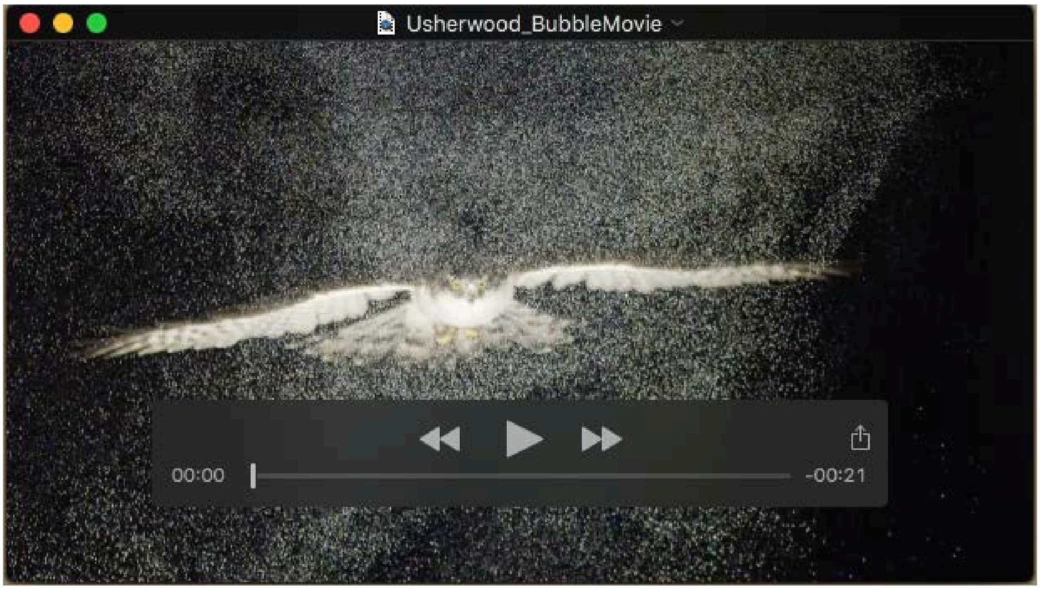

Movie 1

Frontal view video of Goshawk and Tawny Owl passing through illuminated bubble volume. Clips begin at the first frame the volume is illuminated. These views are for context only and were not used in the particle tracking. Recorded at 120 frames per second, shown at 24 fps. Exposure duration 1/140s, or 5/700s, resulting in exactly five 1/7000s strobe flashes each frame. Wingtip trailing vortices persist in the wake, and regions of faster downwash and associated tail tip vortices convect downward following the tail indicating a step increase in lift over the body/tail section.

Journal of Experimental Biology • Supplementary information

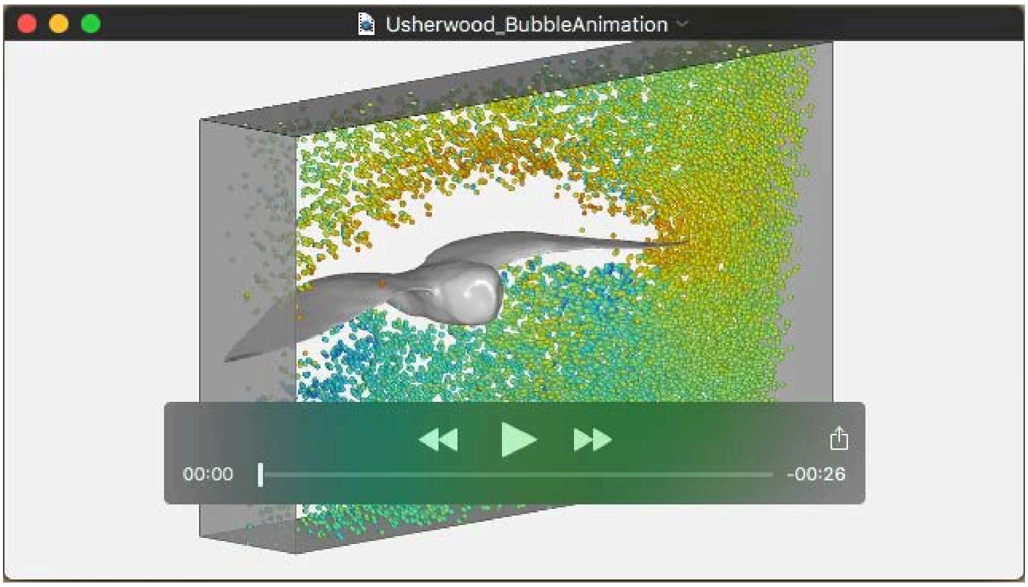

Movie 2

Animation of particles tracked at 700 frames per second, displayed at 30fps for Barn Owl, Tawny Owl and Goshawk. Bird animations derived from stereogrammetry of previous experiments, matched to landmarks measured in the current study. Particle colours indicate vertical velocity, ranging from red upward to blue downward.

Journal of Experimental Biology • Supplementary information
